# Exploring the Cross-Sectional Association between Transit-Oriented Development Zoning and Active Travel and Transit Usage in the United States, 2010–2014

**DOI:** 10.3389/fpubh.2016.00113

**Published:** 2016-06-03

**Authors:** Emily Thrun, Julien Leider, Jamie F. Chriqui

**Affiliations:** ^1^Institute for Health Research and Policy, University of Illinois at Chicago, Chicago, IL, USA; ^2^Division of Health Policy and Administration, School of Public Health, University of Illinois at Chicago, Chicago, IL, USA

**Keywords:** zoning, land use, active travel, physical activity, built environment, policy

## Abstract

**Background:**

In response to traditional zoning codes that contribute to car-dependent sprawling and disconnected neighborhoods, communities are reforming their land use laws to create pedestrian-friendly areas that promote physical activity. One such reform is the adoption of transit-oriented developments or districts (TODs). TODs are higher density, compact, and mixed use areas located around transit stops that are designed to encourage walking.

**Purpose:**

To identify the characteristics of communities that have adopted TODs in their land use laws and examine if communities that have included TODs in their zoning codes are more likely to have adults that commute by any form of active transportation (i.e., walking, biking, or public transportation) or by using public transportation specifically.

**Methods:**

Zoning codes effective as of 2010 were obtained for a purposeful sample of the largest 3,914 municipal jurisdictions located in 473 of the most populous U.S. counties and consolidated cities within 48 states and the District of Columbia. They were evaluated to determine whether they included TOD districts or regulations using a coding tool developed by the study team. Descriptive statistics together with *t*-tests and Pearson’s chi-squared independence test were used to compare characteristics of jurisdictions with and without TOD zoning. Multivariate linear regressions were used to compute the adjusted association between TOD zoning and taking public or active transportation to work.

**Results:**

Jurisdictions with TOD zoning were located more in the South and West than non-TOD jurisdictions and were more populous, higher income, more racially diverse, and younger. Jurisdictions with TOD zoning had significantly higher percentages of occupied housing with no vehicle than those without TOD zoning. TOD zoning was associated with significantly higher rates of public transportation to work (β = 2.10, 95% CI = 0.88, 3.32) and active transportation to work (β = 2.48, 95% CI = 1.03, 3.94).

**Conclusion:**

Communities that have or are considering developing public transit infrastructure may want to modify their zoning codes to include TODs to promote physical activity and active travel to work.

## Introduction

In 2013, ~86% of all workers in the U.S. commuted to work by private automobile, while only 5% used public transportation and ~3% walked or biked to work (1). The U.S. *Surgeon General’s Call to Action on Walking* recently recognized the important role that access to public transit can play in providing people with additional opportunities for walking that may contribute toward their achieving the 30 min of daily physical activity recommended by the *Physical Activity Guidelines for Americans* (2, 3). Public transit users tend to garner additional walking minutes by walking to and from their transit stop or station and walking through public transit stations (2, 4–15). Recognizing the importance of active travel to helping Americans achieve the recommended amounts of daily physical activity, the Surgeon General’s *Call to Action* acknowledges the important role that community design can play in facilitating access to public transportation and locating places where people live, work, and play in close proximity to clean and convenient public transit stops and facilities (2).

The Surgeon General’s *Call to Action on Walking* recognizes the important role that community design and land use can play in facilitating walking opportunities (2). Furthermore, the *Community Preventative Services Task Force* recommends urban design and land use policies as effective strategies for promoting physical activity (16). Local governments regulate urban design and land use policies through their zoning codes, which have been shown to support or inhibit opportunities for physical activity (17–19). It has been recognized that traditional zoning patterns that promote segregated land uses contribute to car-dependent sprawling and disconnected communities (20). In response, jurisdictions nationwide have been reforming their land use laws to create pedestrian-friendly areas to promote physical activity among community members. One such land use reform includes the adoption of transit-oriented developments or districts (TODs) in zoning codes (21). TODs are higher density, compact, and mixed used areas located around transit stops (22). They are designed to be walkable and reduce automobile dependence and encourage the use of public transit and walking or biking as a transportation mode. The density ranges to support a TOD are context dependent and can vary by site. Transit density thresholds can start from <12 housing units per acre for a suburban neighborhood or <20 units per acre for an urban neighborhood (23). Thresholds start from <50 housing units per acre for a suburban center or <60 units per acre for an urban downtown area (23). High density does not necessarily mean the development of high rise buildings. It can also be achieved through small-scale buildings with high lot coverages that create urban design characteristics (e.g., human scale and sense of enclosure) that are supportive to walking (24). The Center for Disease Control’s Transportation Recommendations includes the development of TODs as a policy option for communities to increase access and opportunities for physical activity (25).

Urban design characteristics typical in TODs have been shown to increase levels of active transportation and decrease automobile use. For example, studies have shown a positive relationship between increased density and land use mix and walking or cycling for transportation (26–28). One study found that people drive less when they live in areas that are more walkable (i.e., mixed use and connected street patterns) (29). Another study found that land use mix and street connectivity and access to public transit stops were important motivations for walking for transportation (30). Additionally, adults living in high-walkability neighborhoods had a higher average number of minutes per week of walking for transportation compared to low-walkability neighborhoods (31).

Transit-oriented developments are not a new concept. In fact, they are based off of development patterns from the late nineteenth and the early twentieth centuries when street cars and rail lines were constructed (32). Before the popularity of the automobile, development was concentrated along rail lines and surrounded train stops characterized as walkable and mixed use nodes (32). One early example of a TOD is the Manchester, Altrincham, and South Junction Railway in the United Kingdom, which was planned to support commuter housing for workers in Manchester (33). Copenhagen also has been a leader in TOD development with its 1947 Finger Plan that established five fingers or railway corridors. The areas next to the stations along the corridor were planned to include housing and commercial buildings (33).

Jurisdictions with TODs have helped foster walking and access to transit by various measures. In Atlanta, they facilitate pedestrian access to transit stations by adopting a pedestrian overlay zone that requires developers provide extra sidewalk width or station connections through buildings near station areas (34). In Seattle, Local Improvement Districts were established to generate tax funds to make pedestrian and bike friendly improvements around station areas, and the city worked with the transportation agency to control the supply of parking to encourage transit usage (34). In San Jose, they adopted policies that increase floor area ratios and provide bike parking, and site requirements that orient buildings toward stations near transit areas (34). In this study, we examine the characteristics of communities nationwide that have adopted TODs in their zoning codes, and we examine if communities that have adopted TODs are more likely to have adults who commute to work by using public transportation or any form of active transportation (i.e., walking, biking, or public transportation). To our knowledge, no study has examined the prevalence of the adoption of TODs in zoning codes or the relationship between the adoption of TODs and active transport to work rates at a national level. Based on the urban design concept that TODs are characterized as walkable, compact, and containing mixed uses, we hypothesized that adult active travel to work would be greater in communities that have adopted TODs than in communities that have not.

## Materials and Methods

This cross-sectional study was conducted between May 2012 and June 2015. The University of Illinois at Chicago (UIC) Institutional Review Board deemed that this study did “not involve human subjects” (research protocol #2011-0880).

### Sample

This study was based on a purposeful sample of all municipal jurisdictions located in the most populous 496 counties and 4 consolidated cities in the U.S., representing 76.04% of the U.S. population according to 2010 Census population estimates. The sample frame was restricted to the most populous counties and consolidated cities due to project resources. Based on the authors’ preliminary research that finds zoning primarily occurs at the municipal level, the sample frame was restricted to a census of all 6,438 municipal jurisdictions in these counties and consolidated cities, which collectively cover 49.14% of the U.S. population. Because of resource constraints, we further limited the sample frame to those 4,076 municipalities that represented at least 0.5% of the county/consolidated city population, which only excluded very small municipalities. Collectively, these municipalities covered 96.75% of the full municipal sample frame and 47.54% of the U.S. population. The remaining 28.49% of the U.S. population living in these counties and consolidated cities were located in unincorporated areas or very small municipalities. Twenty-four counties in the initial sample frame contained no municipal jurisdictions, and one consolidated city contained no municipal jurisdiction with at least 0.5% of the total consolidated city population. Thus, the final sample frame included 4,076 jurisdictions located in 472 counties and 3 consolidated cities. (Additional information on sensitivity analyses conducted with the excluded unincorporated areas is presented below under Section “Results.”)

We were unable to obtain the zoning code for 155 of these jurisdictions. Another six jurisdictions had to be excluded from our analysis because we could not obtain data needed to compute our walkability scale, detailed below, and one other had to be excluded because American Community Survey (ACS) data were unavailable. The final analytic sample included 3,914 jurisdictions covering 45.45% of the U.S. population located in 471 of the most populous U.S. counties and 2 consolidated cities, in 48 states and the District of Columbia.

### Data Sources

#### Zoning Codes

Zoning codes were obtained for all 3,914 jurisdictions using primary legal research methods to collect the codes *via* the Internet with 100% telephone or email verification to ensure collection was complete and accurate. To allow a lag for policy implementation, codes with a 2010 effective date were obtained. When the policy had been revised since 2010, we obtained the earlier 2010 version.

#### American Community Survey

Municipal characteristics and measures of taking public transportation or any active transportation (walking, biking, or public transportation) to work were obtained from the Census Bureau’s ACS 2010–2014 5-year estimates (35). The ACS is conducted annually and provides sociodemographic information on each jurisdiction. We used the 5-year estimates as those are the only ones produced for jurisdictions of all sizes, which was necessary as our sample is highly inclusive and is only restricted to municipal jurisdictions comprising at least 0.5% of their county/consolidated city’s population. The 5-year estimates have the added advantage of being the most precise (36).

#### NAVTEQ

ArcGIS 10.1 software was used to access NAVTEQ 2013 data. These data provided counts of all street level intersections and four-way intersections for each jurisdiction, which were used in the construction of the walkability scale described below.

### Measures

#### Active Transport to Work Outcomes

The percentage of workers who worked away from home taking public transportation to work was derived from the ACS 2010–2014 5-year estimates. ACS respondents were asked “How did this person usually get to work LAST WEEK? If this person usually used more than one method of transportation during the trip, mark (X) in the box of the one used for most of the distance.” The response options included car, truck, or van; bus or trolley bus; streetcar or trolley car; subway or elevated; railroad; ferryboat; taxicab; motorcycle; bicycle; walked; worked at home; or other method. The responses bus or trolley bus, streetcar or trolley car, subway or elevated, railroad, and ferryboat were counted as public transportation. We also derived the percentage of workers who worked away from home taking any form of active transportation – including walking, biking, or public transportation – from the ACS 2010–2014 5-year estimates.

#### TOD Zoning

Zoning regulations were coded by Master’s level urban planners using a detailed coding tool and protocol developed by the study team. Each coder was only allowed to code independently after reaching a 90% agreement rate. Two Research Electronic Data Capture (REDCap) databases were developed for tracking policy collection and entering policy coding (37). A dichotomous (yes/no) variable was created to indicate whether each jurisdiction’s zoning code included TOD districts or regulations.

#### Municipal Controls and Walkability Scale

From the ACS data, we derived tertiles of median household income and population size, the percentage of households in poverty, percentage of non-Hispanic White, percentage of non-Hispanic Black, percentage of Hispanic, median age, percentage of occupied housing with no vehicle available, and region. We created a walkability scale using NAVTEQ 2013 and ACS 2010–2014 data to at least partially control for the municipal built environment (resources precluded us from compiling more in-depth measures of the built environment such as presence of sidewalks, crosswalks, bike lanes, etc.). The walkability scale is an additive scale of four density measures: the ratio of four-way intersections to all intersections (NAVTEQ), intersection density computed as the total number of intersections divided by the municipal land area (NAVTEQ), housing unit density (ACS), and population density (ACS). The scale was standardized and adjusted by a factor of 1 to reduce negative scale values. Our scale was based on the previous scale created by Slater and colleagues (38), which in turn was adapted from the scale created and updated by Ewing and Hamidi (39).

### Statistical Analysis

The zoning, ACS, and NAVTEQ data were linked using municipal-level Federal Information Processing Standards (FIPS) geocodes. To compare bivariate differences in continuous characteristics of TOD and non-TOD jurisdictions, *t*-tests were computed without the assumption of equal variances using Satterthwaite’s approximation. To compare bivariate differences in categorical characteristics of TOD and non-TOD jurisdictions, Pearson’s chi-squared independence test was computed. To examine the adjusted association between TOD zoning and taking public or active transportation to work, we computed multivariate linear regressions controlling for the municipal characteristics listed above. The coefficients from these models can be interpreted as percentage point differences in the percentage of workers taking public or active transportation to work. Adjusted prevalences of taking public and active transportation to work with and without TOD zoning were calculated from predictive margins based on these models. Models were clustered on county with robust SEs. Adjusted *R*-squared statistics were computed to assess model fit. All analyses were conducted in Stata S.E. version 13 (40).

## Results

Table 1 shows descriptive characteristics of jurisdictions with and without TOD zoning. Over 5% of jurisdictions had TOD zoning. Rates of workers taking both public transportation and any active transportation to work were two to three times higher in TOD jurisdictions than in non-TOD jurisdictions. Jurisdictions with TOD zoning were located more in the South and West than non-TOD jurisdictions and were more populous than non-TOD jurisdictions. TOD jurisdictions were also more middle and high income than non-TOD jurisdictions. On average, TOD jurisdictions were more racially diverse, with lower percentages of non-Hispanic Whites and higher percentages of non-Hispanic Blacks and Hispanics. TOD jurisdictions were also slightly younger than non-TOD jurisdictions, with an average median age about 2 years lower than that in non-TOD jurisdictions. Notably, TOD jurisdictions had significantly higher percentages of occupied housing with no vehicle on average (9.51% vs. 7.02%, a 35% difference).

**Table 1:** **Characteristics of jurisdictions with and without zoning for transit-oriented development, 2010–2014**.

**Variable**	**% or mean**		***p*-value**
	**TOD jurisdictions (*n*** **= 212)**	**Non-TOD jurisdictions (*n*** **= 3,702)**	
% Workers taking public transportation to work	8.03	2.83	<0.001
% Workers taking active transportation to work	11.97	5.92	<0.001
% Households in poverty	12.61	12.53	0.85
% Non-Hispanic White	54.32	72.15	<0.001
% Non-Hispanic Black	14.91	8.42	<0.001
% Hispanic	19.46	13.24	<0.001
Median age (mean)	36.44	38.38	<0.001
Walkability scale (mean)	1.65	0.97	<0.001
% Occupied housing with no vehicle	9.51	7.02	<0.001
Region			
West	34.43%	18.40%	<0.001
Midwest	8.49%	31.66%	
Northeast	16.98%	22.31%	
South	40.09%	27.63%	
Median household income tertiles			
Low ($17,281.00–$47,434.00)	24.53%	33.87%
Middle (>$47,434.00–$64,924.00)	40.09%	32.93%	0.013
High (>$64,924.00)	35.38%	33.20%
Population size tertiles			
Small (509–6,083)	7.08%	34.85%
Medium (6,084–22,177)	25.94%	33.77%	<0.001
Large (22,178–2,712,608)	66.98%	31.39%

**N* = 3,914 jurisdictions covering 45.45% of the U.S. population located in 471 counties and 2 consolidated cities, located in 48 states and the District of Columbia. For continuous characteristics, *p*-values for the statistical significance of differences are from *t*-tests with no assumption of equal variances computed with Satterthwaite’s approximation. For categorical characteristics, *p*-values are from Pearson’s chi-squared test of independence*.

Table 2 shows the adjusted association between TOD zoning and the percentage of workers taking public and active transportation to work. We find that TOD zoning is associated with a more than 2 percentage point increase in taking public transportation to work (β = 2.10, 95% CI = 0.88, 3.32), and a nearly 2.5 percentage point higher rate of taking any form of active transportation to work (β = 2.48, 95% CI = 1.03, 3.94). These are both large increases: the adjusted prevalence of taking public transportation to work is 3.00% without TOD zoning but 5.10% with TOD zoning, and the adjusted prevalence of taking any form of active transportation to work is 6.12% without TOD zoning but 8.60% with TOD zoning.

**Table 2:** **Adjusted association between zoning for transit-oriented development and % workers taking public or active transportation to work, 2010–2014**.

**Variable**	**% Workers taking public transportation to work**		**% Workers taking active transportation (walking, biking, or PT) to work**	
	**β (95% CI)**	***p*-value**	**β (95% CI)**	***p*-value**
TOD zoning	2.10 (0.88, 3.32)	0.001	2.48 (1.03, 3.94)	0.001
Municipal controls				
% Households in poverty	−0.09 (−0.15, −0.04)	0.001	0.06 (−0.03, 0.14)	0.18
% Non-Hispanic White	−0.12 (−0.19, −0.06)	<0.001	−0.14 (−0.22, −0.07)	<0.001
% Non-Hispanic Black	−0.06 (−0.13, 0.01)	0.07	−0.14 (−0.22, −0.07)	<0.001
% Hispanic	−0.11 (−0.17, −0.04)	0.001	−0.18 (−0.25, −0.10)	<0.001
Median age	0.02 (−0.02, 0.05)	0.29	−0.06 (−0.12, −0.01)	0.016
Walkability scale	1.81 (1.45, 2.17)	<0.001	2.06 (1.64, 2.47)	<0.001
% Occupied housing with no vehicle	0.38 (0.28, 0.47)	<0.001	0.59 (0.48, 0.70)	<0.001
Region				
West	0.50 (−0.25, 1.26)	0.19	1.39 (0.48, 2.31)	0.003
Midwest	0.48 (−0.00, 0.97)	0.051	0.27 (−0.40, 0.94)	0.43
Northeast	2.22 (0.84, 3.59)	0.002	3.01 (1.52, 4.51)	<0.001
South	Referent		Referent	
Median household income tertiles				
Low ($17,281.00–$47,434.00)	−3.27 (−4.02, −2.51)	<0.001	−4.31 (−5.39, −3.22)	<0.001
Middle (>$47,434.00–$64,924.00)	−2.19 (−2.86, −1.51)	<0.001	−2.72 (−3.52, −1.92)	<0.001
High (>$64,924.00)	Referent		Referent	
Population size tertiles				
Small (509–6,083)	0.35 (−0.14, 0.84)	0.16	0.42 (−0.18, 1.01)	0.17
Medium (6,084–22,177)	0.56 (0.01, 1.11)	0.048	0.43 (−0.17, 1.02)	0.16
Large (22,178–2,712,608)	Referent		Referent	
Adjusted *R*^2^	0.53		0.51	
Adjusted prevalence				
Without TOD zoning	3.00		6.12	
With TOD zoning	5.10		8.60	

**N* = 3,914 jurisdictions covering 45.45% of the U.S. population located in 471 counties and 2 consolidated cities, located in 48 states and the District of Columbia. Estimates are from multivariate linear regressions clustered on county with robust SEs, controlling for the variables shown*.

Because the focus of this paper was on municipal zoning, unincorporated areas, where county/consolidated city zoning applies, were excluded from the analyses. As a sensitivity check, we also ran regression models that included those unincorporated areas representing at least 0.5% of the county/consolidated city population. ACS data were not available for unincorporated areas, so we used ACS and NAVTEQ county-level estimates under the assumption that the distribution of characteristics in the unincorporated areas was the same as that in the counties/consolidated cities in which they were located. The results from these models were almost identical to the models presented above: with these unincorporated areas, the estimated *beta* coefficient for the association between TOD zoning and taking public transportation to work was 2.07 (95% CI: 0.91, 3.22) (as compared to 2.10 for the municipal-only models), and the estimated *beta* coefficient for the association between TOD zoning and taking any active transportation to work was 2.45 (95% CI: 1.06, 3.84) (as compared to 2.48 for the municipal-only models). (Additional information on the sensitivity analyses is available from the corresponding author.)

## Discussion

To our knowledge, this is the first national study that examines the characteristics of communities that have adopted TODs in their zoning codes and if the presence of TODs in zoning codes is associated with increased rates of taking public transit to work or engaging in any active transportation to work. The results from this study support the theory that TODs create environments that support physical activity and active transportation to work (21, 25). We found that communities with TOD zoning had significantly higher rates of workers taking public transportation or any active transportation to work than communities without TOD zoning, in both the unadjusted and adjusted models. Previous studies have shown that adult transit users are more physically active than non-transit users (41). Therefore, through the adoption of TODs, a community can support alternatives to automobile travel and potentially increase rates of physical activity, particularly walking (2). Given that this study only examined active transportation to work, and not travel undertaken for other purposes, the overall increase in active travel and physical activity associated with TOD zoning may be even greater than is suggested here.

Interestingly, TOD zoning was present in only 5% of the jurisdictions that we studied. One study that surveyed U.S. developers indicated that even though there is a perceived interest in developing TODs or other pedestrian-friendly developments, there is not enough land supply to implement them due to restrictive land use regulations (42). Developers often face challenges trying to obtain financing for TOD projects due to the perceived risk of mixed use projects, the lack of lenders’ finance experience for TODs, and the fact that TODs are more complex to construct and have a longer build out time than traditional developments (43, 44). Additionally, the lack of TOD implementation may also be due to market conditions related to consumer preference for comparison and one-stop shopping causing larger retailers to locate near each to create an agglomeration of goods and services (45). The space that is provided in a mixed use area might not fit larger retailers making TODs unattractive to them (45).

Finally, there may be political barriers to address when developing a TOD. Rezoning a previously developed area to be supportive of TODs might meet political resistance by local residents who are fearful of change (45). Also, the amount of infrastructure investment to restructure areas to support TODs might be an impediment. Local governments will need to invest in new streets, sidewalks, and other amenities to support this type of development (45). Additional spending might be viewed as wasteful without intended benefits being clearly identified to the public (45). Therefore, it is necessary that local governments facilitate community support and participation to promote development of TODs (43).

Notably, a survey of developers indicated that the presence of supportive zoning was rated the most important factor to affect their decision to develop a TOD in a community (44). Additionally, TOD supportive zoning shows the developer that the community has gone through the planning process and shows that this type of development is supported by the community (44). Changing zoning is a convenient option for local governments who want to attract TODs because it does not involve much expenditure (44).

Interestingly, TOD jurisdictions had younger populations (median age: 36.4 vs. 38.4 years in non-TOD jurisdictions) and had a higher percentage of households without access to a car (9.51% vs. 7.02% in non-TOD jurisdictions). This might be aligned with recent surveys that suggest that a majority of millennials want to live in walkable areas with better transit access and to be less reliant on a car (24, 46). However, the aging baby boomer population is also expressing interest in living in areas that are compact, walkable, and offer increased transportation options (24, 47). They want to be able to age in place, maintain their independence and mobility, and have easy access to amenities.

Additionally, communities with TODs also had higher median incomes than communities without TODs. The establishment of TODs is mostly accomplished by infill development that can be more costly than greenfield development and can drive up property values (48). Since walkable smart growth and New Urbanist communities are in demand, people have been willing to pay a premium to live in them (24). When considering TODs, communities may want to be mindful of lower income residents to ensure that they will not be displaced or face barriers if they want to locate in these areas (48). In fact, TODs can be particularly beneficial for people of low income by allowing residents to live car free and have easy access to jobs, services, and activities (14). Communities could take steps to promote equitable development by incentivizing affordable housing development or creating policies that require affordable housing near transit station areas (48).

However, it is important to note that the adoption of TODs and incentives alone are not sufficient for encouraging developers to construct quality projects near transit areas. Since incentive policies are voluntary, they can often result in inconsistent or uncoordinated urban design standards. Additionally, the incentives offered might not be strong enough to attract developers to an area. Some markets might not have the demand for TOD development. The ability to leverage public funding and joint development finance options to make development more affordable is a stronger mechanism to attract development. Communities within the Washington, DC, area have used master plans as a tool to successfully implement TODs by coordinating development and investments, defining implementation strategies, establishing development standards, and linking projects with other areas (34).

### Study Limitations and Areas for Future Study

This study has several strengths in that it was a national study that examined the characteristics of communities that adopted TODs and the rates of active transportation. However, at the same time, it was subject to several limitations. First, since we collected zoning codes effective as of 2010 to link with our outcome data, there are potentially many more communities in our sample frame and outside of our sample frame that have adopted TODs since our effective date that we did not capture. Future studies should account for any additional communities that have adopted TODs.

Second, because this was a cross-sectional study, the results presented herein should be considered as associations or correlational and not causal – in other words, we can say that TOD zoning is associated with higher rates of public transit use and active travel to work; however, we are unable to say that TOD zoning causes public transit use or active travel to work. Future studies should examine changes within communities over time to examine the longitudinal impact of TOD zoning on active travel to work.

Third, as a cross-sectional study, we were unable to account for the self-selection possibility described in Section “Discussion” – i.e., that people who prefer to live in active-living-oriented environments and participate in active transportation select to live in areas with TODs (24, 46) or if TOD zoning leads to higher rates of active transportation or public transit use. This is an additional area for future study.

Fourth, we were unable to evaluate the transit infrastructure, frequency of transit services, and physical on-the-ground infrastructure within the communities. Future studies should factor this into their analysis to determine if any of these items contribute to people’s decisions to walk, bike, or take public transportation to work. We were also unable to account for the distance workers traveled to their place of employment, an important factor in people’s decisions of how to travel to work that should be factored into future studies.

Fifth, this was a purposeful sample of municipalities located in the most populous counties and consolidated cities in the U.S. We restricted to municipal jurisdictions because most zoning occurs at the municipal level. Sensitivity analyses noted above tested whether we saw a difference in the relationship between TOD zoning and taking public transit or active travel to work when including unincorporated county and consolidated city land areas (as compared to what we saw with the municipal analyses), and the results were nearly identical. Because we were able to capture a nearly complete census of all municipal jurisdictions comprising >0.5% of the county populations, our sample included large and small, geographically, socioeconomically, and demographically diverse municipalities located in 48 states and the District of Columbia. As the first large-scale study of its kind, this was a starting point for understanding the relationship between municipal TOD zoning and adult active travel to work. Future studies may want to examine a random sample of jurisdictions nationwide to assess whether similar results occur and are generalizable to municipalities in the U.S.

Sixth, this study only examined active transportation to work and did not include travel for other purposes such as school, restaurants, or shopping. Future studies should examine the association between TOD zoning and active transportation more broadly.

Finally, future studies may want to explore other benefits of the adoption of TODs such as economic development impacts by providing access to jobs or environmental impacts such as improved air quality by reduced vehicle trips (22). Future studies might also want to examine what specific urban design features (sidewalks, street connectivity, mixed land uses, density, etc.) are desirable and are most likely to create environments conducive to walking, biking, or taking public transit. Additionally, future studies may want to examine the reasoning behind low prevalence of TOD zoning in light of its potential in increasing active transportation.

### Concluding Remarks

The presence of TODs in zoning codes is associated with increased active and public transportation to work rates in a diverse group of communities nationwide. This association suggests that the adoption of TODs may serve as a policy option to increase physical activity; although given the limitations of this cross-sectional study, we cannot establish a causal effect (25). Given the importance of facilitating active travel as a way to promote physical activity, communities may want to consider reforming their land use laws to legalize these types of developments and remove any regulatory restrictions that may make this type of development difficult (48).

## Author Contributions

ET is the lead author of the paper. She was responsible for conducting the literature review, interpreting the results relative to the literature, and drafting and revising the manuscript. JL was responsible for performing analysis of data and assisting with the Methods and Results sections. JC was the investigator for the research grant. She conceptualized the study and this specific analysis, guided interpretation of the results, and substantively reviewed and revised the manuscript content. All authors reviewed the final version of the manuscript that is submitted.

## Conflict of Interest Statement

The authors declare that the research was conducted in the absence of any commercial or financial relationships that could be construed as a potential conflict of interest.
